# Supplementary material for: Mode-specific effects of concentric and eccentric isokinetic training of the hamstring muscle at slow angular velocity on the functional hamstrings-to-quadriceps ratio-a randomized trial
Source: PeerJ. 2022 Sep 29;10:e13842. doi: 10.7717/peerj.13842 (PMC9527019; doi:10.7717/peerj.13842)
Supplement: Supplemental Information 2 [file peerj-10-13842-s002.docx]

Subjects fulfill inclusion criteria

Informed consent, risks, and benefits discussed

Participants allocated to either of the two groups

Concentric isokinetic training group

Eccentric isokinetic training group

Pre-intervention evaluation for PT_ecc_, PT_con,_ AT_hams_, AT_quad_, DT_hams,_ DT_quad_, TPT_hams_, and TPT_quad_

Pre-intervention evaluation for PT_ecc_, PT_con,_ AT_hams_, AT_quad_, DT_hams,_ DT_quad_, TPT_hams_, and TPT_quad_

Intervention:

Concentric isokinetic training of hamstring muscle was performed at 60^0^/sec for 6 weeks.

Intervention:

Eccentric isokinetic training of hamstring muscle was performed at 60^0^/sec for 6 weeks

Post-intervention evaluation for PT_ecc_, PT_con,_ AT_hams_, AT_quad_, DT_hams,_ DT_quad_, TPT_hams_, and TPT_quad_

Post-intervention evaluation for PT_ecc_, PT_con,_ AT_hams_, AT_quad_, DT_hams,_ DT_quad_, TPT_hams_, and TPT_quad_

Data collection

Data Analysis
